# The Building Emotional Awareness and Mental health (BEAM) program developed with a community partner for mothers of infants: protocol for a feasibility randomized controlled trial

**DOI:** 10.1186/s40814-023-01260-y

**Published:** 2023-03-09

**Authors:** Kayla M. Joyce, Charlie Rioux, Anna L. MacKinnon, Laurence Y. Katz, Kristin Reynolds, Lauren E. Kelly, Terry Klassen, Tracie O. Afifi, Aislin R. Mushquash, Fiona M. Clement, Mariette Chartier, Elisabeth Bailin Xie, Kailey E. Penner, Sandra Hunter, Lindsay Berard, Lianne Tomfohr-Madsen, Leslie E. Roos

**Affiliations:** 1 Department of Psychology, University of Manitoba, P314 Duff Roblin Building, 190 Dysart Road, Winnipeg, Manitoba R3T 2N2 Canada; 2 University of Oklahoma, Norman, USA; 3 University of Calgary, Calgary, Canada; 4 Lakehead University, Thunder Bay, Canada; 5 University of British Columbia, Vancouver, Canada; 6 Departments of Psychology and Pediatrics, University of Manitoba, Winnipeg, Canada

**Keywords:** Parenting, Maternal, Mental health, Feasibility, Pilot, Randomized controlled trial, mHealth

## Abstract

**Background:**

Drastic increases in the rates of maternal depression and anxiety have been reported since the COVID-19 pandemic began. Most programs aim to improve maternal mental health or parenting skills separately, despite it being more effective to target both concurrently. The Building Emotional Awareness and Mental health (BEAM) program was developed to address this gap. BEAM is a mobile health program aiming to mitigate the impacts of pandemic stress on family well-being. Since many family agencies lack infrastructure and personnel to adequately treat maternal mental health concerns, a partnership will occur with Family Dynamics (a local family agency) to address this unmet need. The study’s objective is to examine the feasibility of the BEAM program when delivered with a community partner to inform a larger randomized controlled trial (RCT).

**Methods:**

A pilot RCT will be conducted with mothers who have depression and/or anxiety with a child 6–18 months old living in Manitoba, Canada. Mothers will be randomized to the 10 weeks of the BEAM program or a standard of care (i.e., MoodMission). Back-end App data (collected via Google Analytics and Firebase) will be used to examine feasibility, engagement, and accessibility of the BEAM program; cost-effectiveness will also be examined. Implementation elements (e.g., maternal depression [Patient Health Questionnaire-9] and anxiety [Generalized Anxiety Disorder-7]) will be piloted to estimate the effect size and variance for future sample size calculations.

**Discussion:**

In partnership with a local family agency, BEAM holds the potential to promote maternal-child health via a cost-effective and an easily accessible program designed to scale. Results will provide insight into the feasibility of the BEAM program and will inform future RCTs.

**Trial registration {2a}:**

This trial was retrospectively registered with ClinicalTrial.gov (NCT05398107) on May 31st, 2022.

**Supplementary Information:**

The online version contains supplementary material available at 10.1186/s40814-023-01260-y.

## Administrative information

Note: The numbers in curly brackets in this protocol refer to SPIRIT checklist item numbers. The order of the items has been modified to group similar items

### Trial Registration – Data Set {2b}

**Table:** 

**Primary registry and trial identifying number**	ClinicalTrials.gov, NCT05398107
**Date of registration in primary registry**	31 May, 2022
**Primary Sponsor**	University of Manitoba
**Contact for public queries**	LER [Leslie.Roos@umanitoba.ca]
**Contact for scientific queries**	LER University of Manitoba, Winnipeg, Manitoba, Canada
**Public title**	A Community Partnership with Family Dynamics to Examine the Building Emotional Awareness and Mental health (BEAM) Program for Mothers of Young Children
**Scientific titles**	The Building Emotional Awareness and Mental health (BEAM) Program Developed with a Community Partner for Mothers of Infants: Protocol for a Feasibility Randomized Controlled Trial
**Countries of recruitment**	Canada
**Health condition(s) or problem(s) studied**	Depression, Anxiety
**Interventions**	*Intervention*: Building Emotional Awareness and Mental health (BEAM) program
	*Comparator*: MoodMission
**Key inclusion and exclusion criteria**	*Age of eligibility for study:* ≥ 18 years old *Inclusion Criteria:* Identifies as a mother; Has a child 6–18 months old; Resides in Manitoba, Canada; has access to a cellular network; > 9 on the Patient Health Questionnaire-9 and/or Generalized Anxiety Disorder-7; Meets criteria for a major depressive disorder and/or anxiety disorder on the Mini International Neuropsychiatric Interview (MINI); Completed the pre-intervention survey before randomization; Available to attend weekly telehealth sessions; Comfortable with English (spoken and written) *Exclusion Criteria*: Suicide attempt in the past year; Self-harm in the past 6 months; Clinically significant psychotic symptoms, posttraumatic stress disorder, or severe alcohol use disorder on the MINI which would interfere with the mother’s ability to attend and/or benefit from services.
**Study type**	Intervention
	*Allocation:* Randomized *Intervention Model*: Parallel Assignment *Masking*: Unblinded
	*Primary purpose*: Treatment
	Pilot and Feasibility
**Date of first enrolment**	February 2022
**Target Sample Size**	80
**Recruitment status**	Active, not recruiting
**Primary Outcomes**	1. Recruitment Rate 2. Retention Rate 3. Perceived Usefulness 4. Processing Time 5. Post-Intervention Questionnaire Completion Time 6. Follow-Up Questionnaire Time 7. Questionnaire Completion 8. Treatment Adherence 9. Safety
**Secondary Outcomes**	1. Treatment Response 2. Treatment Effect 3. Baseline Severity Moderation
**Demographic Variables**	1. Sociodemographics 2. Adverse Childhood Experiences 3. Program Feasibility and Acceptability

### Protocol Version {3}

February 2023, Version 2

### Name and contact information for the trial sponsor {5b}

Leslie E. Roos, Department of Psychology, University of Manitoba, 190 Dysart Road, Winnipeg, MB, R3B 0S8. Email: leslie.roos@umanitoba.ca.

### Role of Sponsor {5c}

The funding source had no role in the design of this study and will not have any role during its execution, analysis, interpretation of data, or decision to submit results for publication.

### Committee roles and responsibilities {5d}

*Principle Investigators and Co-Investigators:* Study planning; Organization of Parent Advisory Board (PAB); Design and conduct of trial; Preparation of protocol and revisions; Application to ethics review board; Recruitment; BEAM Administration; Reporting of adverse events; Responsible for master file; Budget administration; Data verification; Conduct and interpret analyses; Publication of study reports

*Community Partner, Family Dynamics*: Design of study; Recruitment; BEAM Administration; Interpretation of findings

*Parent Advisory Board (PAB):* Design of trial; BEAM Administration; Interpretation of findings

## Introduction

### Background and rationale {6a–b}

Maternal depression and anxiety have dramatically increased during the COVID-19 pandemic [1]. A meta-analysis documented that 26.9 and 41.9% of mothers experience clinically significant depression and anxiety symptoms, respectively [2]. Key risk factors associated with increased maternal mental health concerns include stressors associated with isolation, domestic conflict, and a lack of parenting support [1, 3]. The absence of standard mental health screening and a backlog of existing mental health services suggests most mothers do not have access to evidence-based mental health treatments [4, 5]. In Canada, only 1 in 10 mothers receive adequate postpartum mental health care, resulting in persistence of problems, enormous individual and societal economic impact, as well as child mental health concerns and developmental impairments [6–9]. The national WeCanForKids report highlighted a need to address maternal mental health even prior to the COVID-19 pandemic [10]. With significant elevations in maternal depression and anxiety compared to pre-pandemic rates [1], there is now a critical need to mitigate the impact of maternal mental health concerns among at-risk families to reduce negative sequelae.

Existing therapies are insufficient to address family mental health needs. Programs designed to improve parent mental health and parenting skills separately have small effects with minimal improvements in the past 20 years [11]. Although programs addressing both mother and child mental health have 50% larger effects than treating either alone, these programs are typically costly, require extensively trained clinicians, and have lengthy waitlists, in turn impeding program scalability and ability to meet maternal mental health needs [12]. Early models of mobile health (mHealth) programs show promise for treating depression and anxiety but are rarely targeted toward mothers and have high dropout rates [13, 14]. Further, mHealth is typically delivered by a third party, without consulting trusted community agencies who hold key knowledge of local needs, can appraise risk, and offer additional support if required.

The Building Emotional Awareness and Mental health (BEAM) program is a mHealth program designed to mitigate the impacts of stress on family mental health and the program’s feasibility is ready to be tested in partnership with an established community agency. The BEAM program was co-designed with a patient advisory board (PAB, i.e., mothers with lived experience managing depression and/or who have completed a previous trial [15]) based on identified priorities of connecting therapists and mothers in a secure online setting. The BEAM platform enables the merger of telehealth best practices with peer connection. Key elements of the BEAM program include the following: (a) expert-led psychoeducational videos adapted from the Unified Protocol (UP), an evidence-based cognitive-behavioral therapy for depression, anxiety, and associated disorders [16–18], as well as emotion-focused parenting strategies [15]; (b) structured telehealth (videoconferencing) group sessions to consolidate therapeutic content and build social support [19, 20]; (c) a monitored online forum to enhance peer connection; (d) symptom monitoring to track progress [21]; and (e) suggested assignments such as reflections and practice exercises. BEAM is therapist-led to increase adherence, consistent with evidence that guided mHealth therapy is more effective than unguided [22]. The BEAM program holds strong potential as a scalable model that can be readily adapted to meet maternal needs, specifically among underserved populations.

Family agencies typically lack the infrastructure and personnel capacities to adequately treat maternal mental health concerns [23], with this gap in services being stark in Manitoba, Canada, where mothers wait 12–18 months for therapy on provincial public health waitlists. At our partner agency, Family Dynamics, parenting coaches find untreated maternal depression and anxiety to be primary in-home risk factors that impede the efficacy of parenting interventions. Providing BEAM training and consultation will enable coaches to provide expert-led mental health treatment to supplement and expand upon existing services. Our partnership approach with Family Dynamics will allow for the assessment of the BEAM program's feasibility when delivered with a community partner.

Our pilot, non-superiority trial of the BEAM program among mothers of toddlers (*N* = 65) found clinically significant change in 66.7% of mothers with persistent depression (anxiety symptoms were not assessed) [24]. Qualitative feedback suggests high acceptability (e.g., “[BEAM] was so helpful in establishing how to accomplish my goal. I also enjoyed getting to know the group via zoom and feel much more comfortable chatting in the forum”; “[BEAM] made me feel less alone in the issues I am facing personally, mental health wise, and in parenting”). This trial was not conducted with mothers of infants and did not include a partnership with an established community agency, however. To inform scalability efforts, further research is needed to determine the feasibility of the BEAM program when delivered in collaboration with a community agency. Following the Obesity-Related Behavioral Intervention Trials (ORBIT) model for developing behavioral treatments for chronic disease [25, 26], our team will complete a feasibility randomized controlled trial (RCT) of the BEAM program. Implementation elements (e.g., maternal depression and anxiety) will be examined to estimate effect size and variance for future sample size calculations and will be compared to a Standard of Care (SOC), i.e., MoodMission. MoodMission was chosen as the SOC as this program has been rigorously studied using RCTs, which is rare for a mHealth app and shows a decrease in moderate depressive and anxiety symptoms post-intervention [27–30].

### Objectives {7}

#### Aim 1

To examine the feasibility of the BEAM program when delivered by a community agency. Feasibility indicators include recruitment rate, retention rate, and perceived usefulness of BEAM, for example.

#### Aim 2

To assess preliminary implementation elements of the BEAM program to estimate effect size and variance for future sample size calculations (treatment effect) and compare the BEAM program to SOC [28] in primary clinical outcomes including maternal depression and anxiety (treatment response).

### Trial design {8}

A two-armed, parallel-design RCT will occur where participants receive the 10-week app-based BEAM program (mental health and parenting treatment) or MoodMission (mental health treatment only). Mothers will be randomized to the BEAM or MoodMission program with a 1:1 allocation as per a computer-generated randomization schedule stratified by participant availability for weekly group telehealth sessions. Self-reported repeated measures data will be collected online via Research Electronic Data Capture (REDCap) [31] from mothers in both programs. Back-end BEAM App data will be collected via Google Analytics and Firebase. Assessments will occur at three timepoints: pre-intervention (T1), post-intervention (T2), and 6-month follow-up (T3). Amendments to the study protocol will be submitted to the Research Ethics Board (REB) at the University of Manitoba who will review and approve amendments prior to implementation. Participants will re-consent to amendments, if required. Any amendments will also be submitted as updates on ClinicalTrials.gov.

This trial is registered with ClinicalTrials.gov (NCT05398107) and will follow Standard Protocol Items: Recommendations for Intervention Trials (SPIRIT) guidelines [32, 33]. All procedures will be performed in accordance with the University of Manitoba’s REB (HE2021-0217) and the Helsinki Declaration. Participants will provide electronic informed consent prior to study enrollment.

## Methods: participants, interventions, and outcomes

### Study setting {9}

Participants will reside in Manitoba, Canada. Manitoba is in the center of Canada and has a population of 1.3 million. Recent results from a predominantly Manitoban sample of mothers with children 0–18 months old suggested significantly elevated rates of depression and/or anxiety since the onset of the COVID-19 pandemic [1].

### Participant identification and consent

Participants will be recruited using online social media advertisements (Facebook, Instagram, Twitter, Pinterest, Reddit), posters in Manitoba, Canada, and poster-sharing via Family Dynamics listserv. Informed consent will be collected electronically from all mothers on three occasions, before (a) the eligibility screener, (b) the MINI [34], and (c) beginning study participation. The consent processes will be documented using REDCap [31]. If funding is obtained for long-term follow-up, further consent will be obtained.

### Eligibility criteria {10}

Participants must (a) identify as a mother (i.e., biological, adoptive, foster, step-mother, or another type of woman-identifying primary caregiver), (b) have a child between 6 and 18 months old at recruitment, (c) be 18 years old or above, (d) reside in Manitoba, Canada, with access to a cellular network, (e) score > 9 on the Patient Health Questionnaire-9 Item (PHQ-9) [35] and/or the General Anxiety Disorder-7 Item (GAD-7) [36], (f) complete a psychodiagnostic mental health assessment (Mini International Neuropsychiatric Interview; MINI) [34] and pre-intervention survey before randomization, (g) meet criteria for a major depressive episode and/or anxiety disorder (i.e., panic disorder, agoraphobia, social anxiety disorder, or generalized anxiety disorder) as assessed by the MINI, (h) be available to attend weekly telehealth groups, and (i) be comfortable understanding, reading, and speaking English. Exclusion criteria include: (a) a suicide attempt in the past year, (b) self-harm in the past 6 months, and/or (c) clinically significant psychotic symptoms, posttraumatic stress disorder, or alcohol/substance use disorder (identified on the MINI 34) which, based on clinical judgement, would make it unlikely for the individual to attend weekly group telehealth sessions and/or make treatment gains. The BEAM and MoodMission programs are not suitable to treat these acute mental health needs and as such mothers deemed ineligible for these reasons will be provided with local mental health resources more appropriate for their needs.

### Participant timeline {13}

See Table 1 for the SPIRIT schedule of enrolment, interventions, and assessments and additional file 1 for the SPIRIT Checklist.

**Table 1:** SPIRIT schedule of enrolment, interventions, and assessments

Study period					
	**Enrolment**	**Allocation**	**Intervention**	**Post-intervention**	**Follow-up**
**TIMEPOINT**	***Weeks 1–8***	**Week 9**	***Weeks 9–19***	***Weeks 19–21***	***Weeks 32–34***
**Informed consent**	X				
**Eligibility screener**	X				
**Psychodiagnostic Mental health Assessment**	X				
**Allocation**		X			
**INTERVENTIONS**					
***BEAM Program***			X		
***Mood Mission Program***			X		
**ASSESSMENTS**					
***Outcome survey***	X			X	X
***Weekly survey***			X		

#### Enrollment (weeks 1–8)

##### Eligibility screener

Mothers will complete an online questionnaire to determine eligibility. This questionnaire will include an author-compiled sociodemographic and suicidality/self-harm questionnaire as well as the PHQ-9 [35] and GAD-7 [36]. These questionnaires will determine whether they meet preliminary inclusion criteria.

##### Psychodiagnostic mental health assessment

If deemed eligible on the eligibility screener, mothers will be invited to complete a psychodiagnostics mental health assessment (i.e., MINI) [34] with trained research personnel via Zoom. The following modules of the MINI will be administered: major depressive episode, suicidality, panic disorder, agoraphobia, social anxiety disorder, posttraumatic stress disorder, alcohol use disorder, substance use disorder, psychotic disorders and mood disorders with psychotic features, and generalized anxiety disorder. Mothers meeting criteria for a major depressive episode and/or an anxiety disorder (i.e., panic disorder, agoraphobia, social anxiety disorder, and/or generalized anxiety disorder) will be deemed eligible once they complete the pre-intervention survey and consent to randomization. Those who do not meet inclusion criteria will be provided with a list of local mental health resources.

##### Pre-intervention questionnaires

All mothers deemed eligible to participate will be invited to complete a series of questionnaires via RedCap to assess baseline data of interest before allocation. See Table 2 for specific questionnaires.

**Table 2:** Schedule for assessment administration

Study period					
**TIMEPOINT**	**Enrolment**	**Allocation**	**Intervention**	**Post-intervention**	**Follow-up**
	***Weeks 1–8***	**Week 9**	***Weeks 9–19***^v^	***Weeks 19–21***	***Weeks 32–34***
**Adverse Childhood Experiences**^**a**^	X				
**Ages and Stages Questionnaire: Social-Emotional, Second Edition** *(6, 12, 18, 24, 30, 36 months)*^b^	X			X	X
**Alcohol Use Disorder Identification Test**^**c**^	X			X	X
**Anxiety Persistence Scale**^a^				X	
**BEAM**^d^** App-Based Questionnaire**^a^				X	
**BEAM**^d^** Forum Questionnaire**^a^				X	
**BEAM**^d^** Perceived Social Support Questionnaire**^a^				X	
**BEAM**^d^** Zoom Telehealth Group Questionnaire**^a^				X	
**Cannabis, Tobacco, and Illicit Drug Use**^a^	X			X	X
**Cannabis Use Disorder Identification Test – Revised**^e^	X			X	X
**Couples Satisfaction Index – 4 Item**^f^	X			X	X
**Depression Persistence Scale**^a^	X				
**Depressive Symptom Index – Suicidality Subscale**^g^				X	X
**Emergency Health & Social Service Utilization**^a^	X			X	X
**Generalized Anxiety Disorder – 2 Item**^h^			X		
**Generalized Anxiety Disorder – 7 Item**^i^	X			X	X
**Infant Behavior Questionnaire – Very Short – Revised**^j^	X			X	X
**mHealth App Usability Questionnaire**^k^				X	
**Mini International Neuropsychiatric Interview**^l^	X				
**Mood Mission App-Based Questionnaire**				X	
**Parenting Scale** *(modified 10 item scale)*^m^				X	X
**Parenting Stress Index**^n^	X			X	X
**Parenting Stress Index** *(modified 4 item scale)*^a^			X		
**Patient Health Questionnaire – 2 Item**^o^			X		
**Patient Health Questionnaire – 9 Item**^p^	X			X	X
**Patient-Reported Outcomes Measurement Information System** *(Anger and Sleep Disturbance Subscales)*^q^	X			X	X
**Pediatric Quality of Life Inventory** *(1–12 or 13–24 months)*^r,s^	X			X	X
**Perceived Maternal Parenting Self-Efficacy Questionnaire**^t^	X			X	X
**Positive and Negative Affect Scale** *(modified 2-item scale)*^u^			X		
**Recent Stressful Experiences**^a^	X			X	X
**Sociodemographics**^a^	X				
**Substance Use Motives Measure** *(Coping Motive Subscales)*^v^	X			X	X

*Note*. ^a^Author-Compiled Questionnaire; ^b^Squires et al. (2015)^c^Babor et al. (2001)^d^BEAM = Building Emotional Awareness and Mental health^e^Adamson & Sellman (2003)^f^Funk & Rogge (2007) ^g^Stanley et al. (2021)^h^Kroenke et al. (2007)^I^Spitzer et al. (2006) ^j^IBQ-VS-R (Putnam et al., 2014) ^k^Zhou et al. (2019) ^l^Sheehan et al. (1998) ^m^Irvine et al. 1999 ^n^Barroso et al. (2016) ^o^Kroenke et al. (2003) ^p^Kroenke et al. (2001) ^q^PROMIS (Hanish et al., 2017; Pilkonis et al., 2011) ^r^Physical Functioning, Physical Symptoms, Emotional Functioning, Social Functioning, and Cognitive Functioning Subscales ^s^Varni et al. (1999) ^t^Barnes & Adamson-Macedo (2007) ^u^Watson et al. (1988) ^v^Biolcati & Passini (2019); ^v^Weekly questionnaires are only administered to those in the BEAM program

#### Interventions (weeks 9–19) {11a}

The BEAM and MoodMission App-based programs will be offered via mobile device (both are functional on Android and iOS platforms). Participants will be provided with a mobile device, if required.

##### The Building Emotional Awareness and Mental Health Program (Intervention Group)

The BEAM program was designed to promote intergenerational well-being through the treatment of maternal mental illness and preventing child mental health concerns, by integrating parenting and self-compassion skills with UP modules [16, 18]. The interrelated skills are designed to increase supportive parenting behaviors and promote maternal-child bonding to disrupt each of the theorized mechanisms of environmental risk transmission (e.g., positive emotional modelling, improved relationships, support for children’s distress, reduced conflict [15, 37]).

The BEAM program is a 10-week guided mental health and parenting program where participants move through modules with a cohort of other mothers experiencing depression and/or anxiety. Each week includes 10–30 min of psychoeducational video content, weekly mood tracking, a 1-hour weekly group telehealth session with content review and facilitated discussions in breakout rooms, access to an online forum to cultivate peer support, and assignments to practice key skills. The BEAM program content has been developed in collaboration with our PAB and the Family Dynamics team.

###### Clinical team

BEAM clinical coaches will consist of mental health therapists (completing a Master's or Doctoral degree in Clinical Psychology who are supervised by a registered clinical psychologist), parent coaches from Family Dynamics, and a peer coach. Parent coaches are mental health staff (trained paraprofessionals) from Family Dynamics. Peer coaches are mothers who recently completed a program targeting their own mental health needs and who have agreed to help promote maternal mental wellness [15]. Clinical coaches will lead/co-lead weekly group telehealth sessions, manage participant contact via email/phone, and participate in weekly supervision meetings with clinical psychologists. Parent and peer coaches will participate in weekly group telehealth sessions and engage with participants on the online community forum.

###### Psychoeducation

The BEAM program will include psychoeducational materials consisting of separate weekly videos on mental health and parenting skills. These videos will be approximately 5 to 15 min long each (totaling 10 to 30 min per week). Mental health videos will provide easily digestible information on emotion regulation strategies adapted from the UP, an evidence-based transdiagnostic treatment for depressive and anxiety disorders [16, 18], and will focus on self-compassion skills. Parenting skill videos will teach emotion-focused and behavior management strategies to help mothers understand and co-regulate their child’s challenging behaviors. Videos will also teach mothers how to prevent negative mother–child interactions and promote positive relationships.

###### Mood tracking

Brief weekly questionnaires on depression (Patient Health Questionnaire-2 Item; PHQ-2) [38], anxiety (Generalized Anxiety Disorder-2 Item; GAD-2) [39], parenting stress (modified version of the Parenting Stress Index; PSI-4) [40], and positive mood (modified version of the Positive and Negative Affect Scale; PANAS-2) [41] will be sent to participants. Scores for each questionnaire will be provided, allowing mothers to track their weekly scores. Mood tracking is shown to promote emotional understanding [42].

###### Weekly group telehealth sessions

Weekly group telehealth sessions will provide mothers with the opportunity to discuss program material with coaches and peers, ask questions, and gain a sense of community. There will be three telehealth groups with different schedules to accommodate as many mothers as possible.

###### Online community forum

An online community forum will provide mothers with the opportunity to reflect on learned skills from the BEAM weekly modules and access social support by connecting with other mothers in the program. This will be a confidential, closed forum overseen by peer and parent coaches. The forum will consist of open-ended discussions between participants, peer advice, and sharing of anecdotes/non-identifying photos of their wellness journey. Mothers will be able to ask parent and peer coaches mental health- and parenting-related questions.

##### MoodMission (Standard of Care Group)

The SOC arm will receive MoodMission, an App-based program effective in the treatment of moderate depression and anxiety in adults and increasing their well-being [27–30]. MoodMission uses an adaptive learning algorithm based on self-reported level of distress during prior engagement. Based on this algorithm, participants are provided with five targeted cognitive-behavioral therapy strategies to build self-efficacy. Compared to BEAM, this self-paced generalized service is not tailored to the complex needs faced by parents and lacks coaching, social support, and parenting skills development. MoodMission was chosen as the SOC because it has been rigorously studied using RCTs [27–30]. Those in the MoodMission group will work through program content on the App at their own pace.

#### Post-intervention questionnaires (weeks 19–21)

Mothers will be given 2 weeks to complete the post-intervention questionnaires via RedCap after the BEAM and MoodMission programs are complete (see Tables 1 and 2). Participants will be invited to complete these questionnaires regardless of program completion.

#### Follow-up questionnaires (weeks 32–34)

Approximately 6 months following program completion, mothers will be sent a series of follow-up questionnaires via RedCap (see Tables 1 and 2).

#### Compensation

Mothers will be compensated a total of $160/Canadian Dollars ($50 for enrollment [$25 for a psychodiagnostic mental health assessment and $25 for pre-intervention surveys], $50 for the post-intervention questionnaires, $25 for the follow-up questionnaires, $25 for participants who complete > 75% of the weekly surveys, and $10 for those who complete the post-intervention questionnaire within 1 week of distribution)^1^ Participants deemed ineligible after the psychodiagnostics mental health interview will be compensated with $25/Canadian Dollars.

### Criteria for discontinuing or modifying allocated interventions {11b}

Clinical coaches will receive an alert from REDCap [31] if suicidality items are endorsed during the pre-intervention, post-intervention, or follow-up surveys [i.e., endorsing “Thoughts that you would be better off dead or of hurting yourself in some way” on the PHQ-9 [35] or a 2 + on any items on the Depression Severity Index—Suicidality Subscale (DSI-SS) [43]] and/or during the psychodiagnostics mental health assessment. These alerts will prompt clinical coaches to take necessary steps to assess participant safety and provide appropriate intervention. The clinical team will consult on any instances of suicidality to determine whether it is in the participant’s best interest to continue in the study. Of those in the BEAM program, participants may have discontinued access to the online community forum and/or denied access to weekly group telehealth sessions if they repeatedly violate terms of use. Further, if a participant cannot maintain the confidentiality of others during weekly group telehealth sessions, they may be denied access. In the above case, participants will still have access to all psychoeducational materials on the BEAM app and will be invited to complete all questionnaires. Clinical coaches would also provide referrals to other clinicians and clinics, if appropriate.

### Strategies to improve adherence and intervention protocols {11c}

Once a mother is randomized, all efforts will be made to follow the participant for the study duration. A recent iteration of BEAM for mothers of children 18–36 months old had an 83.7% retention rate (16.3% lost to follow-up) [44]. First, mothers will be asked to confirm their availability for weekly group telehealth sessions on the eligibility screener and will participate in a Zoom orientation with a parent coach. Second, participants in the BEAM program will receive three weekly push notifications via the BEAM App as well as a weekly reminder via email. Engagement notifications contribute to increased mental health benefits of mHealth interventions [45]. Third, those in the BEAM program will also be contacted by their clinical coach if they have not attended a weekly telehealth group session. Fourth, compensation will be provided across study participation (see below) to encourage completion; participants will be encouraged to complete questionnaires regardless of program completion.

### Outcomes {12}

Pre-specified success criteria are identified in Table 3. All feasibility indicators will be dichotomized into success or revise. “Success” indicates the protocol can move forward with a larger RCT with no/little revisions required, while “revise” suggests changes are needed prior to proceeding.

**Table 3:** Feasibility indicators

Feasibility	Indictor	Criteria for success
***Aim 1***		
Recruitment rate	Number of mothers per week recruited	Mean 2 participants/week
Retention rate	% of mothers with data at follow-up	> 80% of mothers with follow-up data
Perceived usefulness	Usefulness subscale of mHealth App Usability Questionnaire	Mean score of < 3
Processing time	Time from initial contact until study enrollment	Mean time is < 1 week
Post-intervention questionnaire completion time	Time to complete post-intervention questionnaires	Mean of < 60 min to complete
Follow-Up questionnaire completion time	Time to complete follow-up questionnaires	Mean of < 60 min to complete
Questionnaire completion	% of missing data among program completers	> 80% of data is present among mothers who completed follow-up
Treatment adherence	Logging onto BEAM App and/or attending weekly group telehealth session	Participation in > 50% of program weeks via logging onto BEAM App or attending weekly group telehealth session
Safety	Adverse events from the BEAM program or assessments	No adverse events reported
***Aim 2***		
Treatment response	Comparison between groups	Significant group difference in primary clinical outcomes (depression and anxiety)
Treatment effect	Estimate of effect size and variance for future sample size calculations	Data on all relevant variables
Baseline severity moderation	Comparison of those with low versus high baseline scores on the PHQ-9 and GAD-7	Significant group difference

#### Aim 1

Back-end App data, collected via Google Analytics and Firebase, will be used to assess recommended mHealth engagement variables [46, 47]: (a) number of logins and sessions to the BEAM app, (b) number of forum posts, (c) time spent on the BEAM app and on the forum, (d) number of videos watched/time spent watching videos, and (e) number of weeks between first and last engagement with the app. Weekly telehealth group session attendance (yes/no) will be compiled by the clinical team. Analytics and group attendance data will also be used to assess retention (number of weeks completed) and completion (participating in > 50% of program weeks by either logging onto the BEAM app or attending the weekly group telehealth session). Finally, the mHealth App Usability Questionnaire (MAUQ) [48] will assess participants’ views on the BEAM program.

#### Aim 2

Data on maternal depression (PHQ-9) and anxiety (GAD-7) will be used to estimate effect size for a future large-scale RCT and to assess preliminary mean change in variables of interest across assessment timepoints. The PHQ-9 includes [35] nine items which will ask mothers the severity of their depressive symptoms during the past 2 weeks. The PHQ-9 has high internal consistency (*α* = 0.88) [49]. Anxiety symptom severity will be assessed using the GAD-7, a seven-item measure which will ask mothers their degree of anxiety symptoms over the past 2 weeks. The GAD-7 has excellent internal consistency (*α* = 0.92) [36]. Items on both scales are scored on a 4-point Likert scale ranging from 0 (“Not at all”) to 3 (“Nearly every day”) where higher summative scores suggest more problematic symptoms.

Additional questionnaires will assess maternal mental health (i.e., Patient Reported Outcomes Measurement Information System [PROMIS] Anger [PROMIS-A] [50] and Sleep Disturbance [PROMIS-SD] [51] subscales, Alcohol Use Disorder Identification Test [AUDIT] [52] Cannabis use Disorder Identification Test – Revised [CUDIT-R] [53], Substance Use Motives Measure—Coping Subscales [SUMM] [54], and Depression Severity Index—Suicidality Subscale [DSI-SS] [43]), parenting (i.e., Parenting Stress Index – Short Form [PSI-SF] [40], Perceived Maternal Parenting Self-Efficacy [PMP S-E] [55], and the Parenting Scale—Overreactivity Subscale [56], Couples Satisfaction Inventory 4-Item [CSI-4] [57]), and child outcomes (i.e., Ages and Stages Questionnaire: Social-Emotional Challenges – 2 [ASQ:SE-2] [58], Pediatric Quality of Life Inventory [PedsQL] [59], and Infant Behavior Questionnaire – Revised – Very Short Form—Effortful Control Subscale [IBQ-R-VS] [60]).

Data on sociodemographic characteristics will be collected in the pre- and/or post-intervention questionnaires including (a) maternal demographics (e.g., age, marital status, highest level of education, ethnicity, employment, depression persistence, anxiety persistence), (b) child demographics (e.g., child sex, age of child), and (c) household demographics (e.g., number of adults and children in the household, annual household income, community type).

Other variables known to impact adult mental health and child development will be collected including the Adverse Childhood Experiences (ACEs) Questionnaire, the author-compiled Recent Stressful Experiences Questionnaire (RSE; developed based on recommendations from the JPB research network on toxic stress at Harvard’s Center on the Developing Child) [61], and the author-compiled Emergency Health and Social Service Utilization Questionnaire.

### Tracking of concomitant care during the trial {11d}

Participants will be permitted to receive concomitant care during study involvement. Concomitant care (e.g., medication, psychotherapy) will be measured during the post-intervention and follow-up surveys (i.e., “During the past 3 months, did you access any other mental health and/or parenting resources?”, “What resources did you access for mental health and/or parenting?”, and “How effective do you think these resources were?”).

### Sample size {14}

As this is a pilot and feasibility study, a sample size calculation is not appropriate. However, our target sample size of 30 participants per group is consistent with prior recommendations and will provide data to sufficiently establish BEAM feasibility [62, 63]. Accounting for a conservative 30% attrition rate [24], we will recruit an additional 10 participants per group (*N* = 80). With an estimated BEAM sample size of 40 mothers, we aim to achieve questionnaire completion and retention rates of 80% to within a 95% confidence interval of ± 12.4% as well as a treatment adherence rate of 50% to within a 95% confidence interval of ± 15.5%.

## Methods: assignment of interventions

### Allocation {16a–c}

Mothers will be randomized to the BEAM or MoodMission program with a 1:1 allocation. Randomization will occur in blocks of 10 using a computer-generated randomization schedule (http://www.randomlists.com/team-generator) stratified by participant availability for weekly group telehealth sessions. To conceal allocation sequence, randomization will occur once 10 mothers have been deemed eligible.

### Blinding {17a–b}

A research assistant unaffiliated with the BEAM clinical team will be responsible for conducting central randomization for participants who have agreed to participate and have received their identification number (i.e., following completion of pre-intervention questionnaires). The enrollment of mothers to the proposed study and their allocation to BEAM versus SOC will be completed by two separate research assistants. The research assistant designating treatment condition will have no interaction with participants (i.e., not on the BEAM clinical team). Trial participants, clinical coaches, parent coaches, and peer coaches will not be blinded to treatment condition given the behavioral nature of the treatment provided. Data analysts also will not be blinded to treatment condition.

## Methods: data collection, management, and analysis

### Data collection methods {18a–b}

An outline of all questionnaires to be used is presented in Table 2. Once a mother is randomized, all efforts will be made to follow the participant for the study duration. A recent iteration of BEAM for mothers of toddlers had an 83.7% retention rate [65], suggesting high retention rates are likely. Since this RCT will include an intent-to-treat analysis, participants will be encouraged to complete questionnaires regardless of program completion. Participants may withdraw from the study at any time, and the investigators may withdraw a participant to protect them and/or others in the program or if the study is terminated prior to the planned end date.

### Data management {19}

Participants will enter questionnaire responses in REDCap [31] which is managed by the Centre for Healthcare Innovation in Winnipeg, Manitoba, who are consultants on the proposed project. An administrative backup for roll-back purposes will occur daily, and data will be exported weekly and housed on an encrypted drive. Data quality checks will be conducted prior to analyses (e.g., range checks for data values). Data will be stored on a secure server in accordance with policies of the University of Manitoba’s REB and Personal Health Information Act (PHIA) policies.

### Statistical methods {20a}

All analyses will be conducted using IBM Statistical Package for the Social Sciences (SPSS). Descriptive statistics will be computed for the total sample and between the two groups (i.e., BEAM and MoodMission) for demographics and outcome measures at pre-intervention.

#### Aim 1

Descriptive statistics from back-end App data, telehealth group attendance, and questionnaires (i.e., BEAM App-Based, BEAM Forum, BEAM Perceived Social Support, BEAM Zoom Telehealth Group, mHealth App Usability, and MoodMission App-Based) will be used to examine program feasibility, engagement, and acceptability.

#### Aim 2

Data on maternal depression (PHQ-9) [35] and anxiety (GAD-7) [36] will be used to estimate effect size and variance for future sample size calculations. Preliminary analyses will also examine treatment effects on outcomes of interest (e.g., depression, anxiety). A longitudinal analysis of covariance, using linear mixed modelling, will be conducted to preliminarily test treatment effects on outcomes of interest. Treatment effects will be tested using two-way interactions between time and group assignment. Baseline moderation by symptom severity will be assessed to determine whether the BEAM program helps reduce symptoms for mothers with higher levels of depression and anxiety at pre-intervention compared to MoodMission. An *α* of 0.05 will determine statistical significance. Aggregate variables will be computed, by converting each measure to a standardized *z*-score then taking the average. Results will be reported as means and 95% confidence intervals for all comparisons. Bonferroni corrections are considered very conservative [64], and it has been suggested that correction may not be necessary, particularly if the study purpose is exploratory [65].

### Additional analyses {20b}

A separate subgroup analysis will be conducted with mothers who report at-risk substance use. This analysis will assess outcomes among mothers with at-risk substance use in the BEAM program and MoodMission. Between- and within-group differences will be examined among mothers with at-risk substance use (an 8 + on the AUDIT and/or CUDIT-R^52^,^53^) and non-problematic substance use (< 8 on the AUDIT/CUDIT-R^52^,^53^). An examination of demographics on feasibility data and a longitudinal analysis of covariance (as described above) will also be conducted.

### Definition of analysis population related to protocol non-adherence and methods to handle missing data {20c}

An intent-to-treat analysis will be conducted where missing data will be handled using full information maximum likelihood estimation [66]. Sensitivity analyses will be conducted by re-running analyses with completers only [67].

## Methods: monitoring

### Composition of the data monitoring committee, its role and reporting structure {21a}

The current trial does not require a formal data monitoring committee given the minimal risk, short duration, and small sample size. The trial will be monitored by the primary investigators who will review each phase of the trial including recruitment, randomization, intervention, and data collection. Weekly meetings will occur with all clinical and research personnel to discuss and resolve questions/concerns and ensure consistent implementation.

### Interim analyses and stopping guidelines {21b}

No interim analyses are planned.

### Plans for collecting, assessing, reporting, and managing adverse events and harms {22}

No adverse experiences have occurred during the prior BEAM pilot study [24]. If participants report suicidality and/or self-harm risk, the clinical team will be notified. A risk management protocol will be in place where follow-up will occur among those identifying suicidality and/or self-harm. Clinical coaches will also follow-up with participants who identified suicidality on the online community forum and/or in weekly group telehealth sessions. The University of Manitoba’s standard procedures for reporting adverse events and protocol violations/deviations will be followed.

### Frequency and plans for auditing trial conduct {23}

It is possible that the University of Manitoba Research Ethics Board may request an audit; however, the study team has no plans to conduct an independent audit.

## Ethics and dissemination

### Research ethics approval {24}

The protocol for this RCT has been approved by and will be performed in accordance with the University of Manitoba’s REB (HE2021-0217).

### Protocol amendments {25}

Amendments to this protocol will be submitted to the University of Manitoba’s REB who will review and approve amendments prior to implementation. Participants will re-consent to amendments, if required. Amendments will also be submitted as updates on ClinicalTrials.gov.

### Confidentiality {27}

The BEAM program will be provided on a secure platform. Participant confidentiality will be protected throughout the study in accordance with the University of Manitoba’s REB guidelines, with exceptions to confidentiality including suspected cases of child maltreatment (abuse, neglect, or endangerment) and imminent harm to self or others. Only specific individuals will have access to data who are trained in the University of Manitoba’s ethics and data safety protocols, have completed PHIA training, and have taken an oath of confidentiality.

All data, including identifying information, will be password protected and stored on REDCap [31]. All data will be associated with a de-identified participant identification number. A password-protected master list will be kept containing the first names and last initials, email addresses, phone numbers, and group allocation of participants. This master list will only be accessible by a research assistant.

Precautions will be taken to manage confidentiality on the online community forums which will include a Data Management Protocol, participant agreed upon Terms of Use (i.e., no personally identifying/health information, no identifiable photographs). Coaches will have the ability to remove posts which violate the terms of use and participants will be emailed should any concerns arise. Security measures also include monitoring of the forums by the study team and the ability for other participants to anonymously flag posts for review.

During the weekly telehealth group sessions, participant anonymity cannot be guaranteed. Telehealth sessions will be hosted on a secure Zoom Healthcare account which is password-protected. At the first group session, the clinical coaches will outline limitations of confidentiality and anonymity. Participant attendance and client records will be tracked through the Titanium platform, hosted by the University of Manitoba Psychological Service Centre, which is PHIA-compliant.

### Declaration of interests {28}

The authors of this RCT declare no conflicts of interest.

### Access to data {29}

All data will be housed in a secure database accessible by the study investigators.

### Ancillary and post-trial care {30}

If it is determined that a mother requires further services, they will be referred to a clinician or clinic, and provided with a list of mental health resources.

### Dissemination policy {31a}

Research findings will be disseminated to academic and public audiences. Several publications describing the feasibility of the BEAM program will be published in peer-reviewed journals following Consolidated Standard of Reporting Trials (CONSORT) guidelines and the CONSORT extension for pilot and feasibility trials [68, 69]. Manuscripts will be posted rapidly on preprint servers, such as psyarxiv.com, and open access (through publishers and/or repositories) for a wider reach. The study investigators and research trainees will present findings at national and international conferences. Our knowledge mobilization plan follows an integrated knowledge translation approach [70]. At the patient level, we will employ multiple strategies to understand and address the unique stressors relevant to maternal depression and anxiety. The PAB is committed to dissemination efforts by contributing to lay summaries, infographics, and sharing in parenting groups/forums. Our agency partner, Family Dynamics, will distribute results with their networks. Collaborating agencies, i.e., Public Health Agency of Canada, The Department of Families, and Canadian Perinatal Mental Health Collaborative, will aid in the sharing of results to network funders, boards, and policy makers. Qualitative focus group interviews with participants and coaches are planned to gain information and to inform program improvements.

### Authorship eligibility guidelines {31b}

Individuals will be considered eligible for authorship if they contributed to the design, conduct, analysis, or interpretation of the data. Professional writers will not be used for manuscript preparation.

### Plans to give access to the full protocol, participant-level data, and statistical code {31c}

De-identified data may be made available on public data platforms such as the Open Science Framework or if made a requirement by the granting agency, journal, or community partner. Statistical code used to conduct analyses for published manuscripts may also be included on public data platforms, allowing other researchers to assess the studies’ scientific rigor.

## Discussion

BEAM is an app-based maternal mental health program which is easily accessible and was designed to scale. The program targets peer connection, psychoeducation, and intervention by mental health professionals and has the potential to mitigate the negative impacts of maternal mental health concerns and stress on family well-being during the COVID-19 pandemic and beyond. The proposed RCT will build upon our prior research on the BEAM program [24] through partnership with a local community agency, Family Dynamics, as agencies commonly lack infrastructure and personnel to adequately treat maternal mental health concerns. In partnership with family agencies, BEAM holds the potential to promote maternal-child mental health through the provision of mHealth content, training, and consultation. Findings will provide information on the feasibility, engagement, and accessibility of the BEAM program and will inform future RCTs of the BEAM program.

### Trial status

Recruitment began in January 2022 and finished in March 2022. The date of first enrollment was February 14th, 2022. The intervention launched in April 2022 (with estimated completion in December 2022 for the collection of all primary outcomes).

## Supplementary Information

**Additional file 1.**

## Data Availability

All data will be housed in a secure database accessible to the study investigators.

## Abbreviations

ACE: Adverse Childhood Experiences
ASQ:SE-2: Ages and Stages Questionnaire: Social-Emotional Challenges – 2
AUDIT: Alcohol Use Disorder Identification Test
BEAM: Building Emotional Awareness and Mental health
CSI-4: Couples Satisfaction Inventory – 4 Item
CUDIT-R: Cannabis Use Disorder Identification Test – Revised
CONSORT: Consolidated Standard of Reporting Trials
DSI-SS: Depression Severity Index—Suicidality Subscale
GAD-2: Generalized Anxiety Disorder – 2 Item
GAD-7: Generalized Anxiety Disorder – 7 Item
IBQ-R-VS: Infant Behavior Questionnaire – Revised – Very Short Form
MAUQ: MHealth App Usability Questionnaire
mHealth: Mobile Health
MINI: Mini International Neuropsychiatric Interview
ORBIT: Obesity-Related Behavioral Intervention Tool
PAB: Patient Advisory Board
PANAS-2: Positive and Negative Affect Scale
PedsQL: Pediatric Quality of Life Inventory
PHIA: Personal Health Information Act
PHQ-2: Patient Health Questionnaire – 2 Item
PHQ-9: Patient Health Questionnaire – 9 Item
PMP S-E: Perceived Maternal Parenting Self-Efficacy
PROMIS: Patient Reported Outcomes Measurement Information Systems
PROMIS-A: Patient Reported Outcomes Measurement Information Systems – Anger Subscale
PROMIS-SD: Patient Reported Outcomes Measurement Information Systems – Sleep Disturbance Subscale
PSI-4: Parenting Stress Index – 4 Item
PSI-SF: Parenting Stress Index – Short Form
RCT: Randomized Controlled Trial
REB: Research Ethics Board
REDCap: Research Electronic Data Capture
RSE: Recent Stressful Experiences
SOC: Standard of Care
SPIRIT: Standard Protocol Items: Recommendations for Intervention Trials
SPIRIT-PRO: Standard Protocol Items: Recommendations for Intervention Trials – Patient Reported Outcome
SPSS: Statistical Package for the Social Sciences
SUMM: Substance Use Motives Measure
T1: Pre-intervention
T2: Post-intervention
T3: 6-Month follow-up
UP: Unified Protocol

## Footnotes

1 Participants in the BEAM program will be compensated $25 for completing > 75% of the weekly symptom tracking surveys, while those in MoodMission will receive an additional $25 as they are not required to complete weekly surveys.
